# The chain mediating roles of anxiety and depression in the relationship between the effects of the COVID-19 pandemic and procrastination in adolescents: a longitudinal study

**DOI:** 10.1186/s12889-023-16605-8

**Published:** 2023-11-17

**Authors:** Zhengxue Qiao, Yongmei Wu, Yunjia Xie, Xiaohui Qiu, Lu Chen, Jiarun Yang, Hui Pan, Simeng Gu, Xiuxian Yang, Xiaomeng Hu, Ping Wei, Jinxin Zhao, Yuanpeng Qu, Jiawei Zhou, Tianyi Bu, Yanjie Yang

**Affiliations:** 1 Psychology and Health Management Center, Harbin Medical University, No.157, Baojian Road, Nangang District, Harbin, 150081 China; 2 Beijing Hospital, Beijing, China; 3 Department of Psychology, School of Education of Heilongjiang University, Heilongjiang Province, Harbin, China; 4 Department of Endocrinology, Peking Union Medical College Hospital, Beijing, China; 5 Department of Psychology, Jiangsu University Medical School, Zhenjiang, Jiangsu Province, China; 6 School of Western Languages and Cultures, Harbin Normal University, Heilongjiang Province, Harbin, China

**Keywords:** Structural equation modelling, Longitudinal studies, Adolescence, Anxiety, Depression

## Abstract

**Background:**

The relationship between the Coronavirus Disease 2019 (COVID-19) pandemic, which is a traumatic event for adolescents, and procrastination is not clear. Mental health may play an important role in this relationship; however, the underlying mechanisms remain unknown. This study aimed to construct chain mediation models to examine whether anxiety and depression symptoms mediate the effects of the COVID-19 pandemic on procrastination in adolescents.

**Methods:**

A convenience sample of 12 middle and high schools in Harbin, China, with four follow-up online surveys was conducted during the COVID-19 pandemic. A total of 4,156 Chinese adolescents were enrolled in this study, of whom ages 11–18 (Mean = 13.55; SD = 1.18), 50.75% were male, and 93.24% were middle school students. Descriptive demographic analysis and Pearson’s correlation analysis of the effects of the COVID-19 pandemic (T1), anxiety(T2), depression (T3), and procrastination (T4) were performed in SPSS 22.0. Chain mediation analysis performed with Mplus 8.3.

**Results:**

The effects of the COVID-19 pandemic, anxiety symptoms, depression symptoms, and procrastination were positively correlated (*P* < 0.01). The effects of the COVID-19 pandemic have a direct link on adolescent procrastination (effect = 0.156; SE = 0.031; 95%CI: 0.092, 0.214), and have three indirect paths on procrastination: the independent mediating role of anxiety symptoms was 29.01% (effect = 0.047; SE = 0.012; 95%CI: 0.024, 0.072), the independent mediating role of depression symptoms was 29.01% (effect = 0.047; SE = 0.010; 95%CI: 0.030, 0.068), as well as the completely chain mediating role of anxiety and depression symptoms was 15.43% (effect = 0.025; SE = 0.005; 95%CI: 0.017, 0.036).

**Conclusions:**

Our results suggest that anxiety and depressive symptoms are part of a causal chain between the effects of the COVID-19 pandemic and procrastination among Chinese adolescents. To effectively reduce their procrastination, attention should be paid to the emotional distress caused to adolescents by major events such as the COVID-19 epidemic. All data were taken from self-reported measures and one city in China, which may bias the results and limit their generalizability.

## Background

Procrastination, the psychological concept of voluntarily or habitually delaying unpleasant tasks until a later time, characterized by short-term pleasure and long-term disadvantages [1–3]. Approximately 75% of college students consider themselves to be procrastinators, nearly half whom procrastinate consistently and problematically [4]. A meta-analysis by Lu et al. has established that procrastination is prevalent across broad sociodemographic conditions and traits [5]. All people tend to procrastinate at various times in their lives [6]. However, adolescence is a particularly vulnerable period of development characterized by a still-maturing control and coping abilities [7]. Adolescence and young adulthood are the most vulnerable to procrastination, with the figure reaching up to 95% of adolescents [4], as well as the desire to overcome it [8]. Procrastination significantly affects human health, well-being, work efficiency and academic performance [9]. Given the negative effects that procrastination can have on academic achievement and well-being, it becomes critical to understand the factors that drive procrastination in adolescents in order to target interventions.

COVID-19 is a health threat that has been identified as a significant stressor that threatens the physical and mental health of individuals around the world. Most studies on this topic have concluded that isolation, online learning, and other measures during the pandemic have increased procrastination among students [10, 11]. Indeed, according to ego depletion theory, prolonged exposure to stress adversely affects an individual's subsequent performance of self-regulatory behaviors, leading to procrastination. Changes in academic lifestyles and heightened uncertainty about the future following the COVID-19 outbreak have made procrastination common among adolescents. Deng et al. showed that COVID-19-related emotional stress was positively associated with bedtime procrastination in college students. Despite the prevalence of procrastination during the pandemic, there are no studies in the literature on the link between the effects of COVID-19 and adolescent procrastination. Therefore, based on ego depletion theory and empirical evidence, we can hypothesize that COVID-19 affect positively predicts adolescent procrastination.

The COVID-19 pandemic and related measures have posed a major threat to mental health [12]. From the perspective of stress response mechanisms, individuals who are stimulated by stress may trigger related emotional responses. According to a meta-analysis performed during the COVID-19 pandemic, depression and post-traumatic stress disorder (PTSD) were the most serious emotional disorders, and approximately one-quarter of people felt anxious, thus potentially indicating considerable social pressure [13]. Adolescents may be particularly vulnerable to the psychological effects of the COVID-19 pandemic [14]. Loades et al. have shown that children and adolescents may be more likely to exhibit high rates of depression and anxiety during and after mandatory isolation (e.g., quarantine) [15].

In addition, emotions play a decisive role in human behavior and decision-making processes. Negative emotions may lead to maladaptive behaviors such as procrastination. According to construal level theory, negative emotions lead people to seek immediate full enjoyment and neglect the pursuit of long-term goals or higher achievements [16]. As a result, people experiencing negative emotions may give up on completing difficult tasks or postpone them for as long as possible. In addition to this, procrastination, as a typical failure of self-regulation, can be considered as a dysfunctional state-of-mind regulation strategy [17]. Individuals experiencing negative emotions can repair their bad mood by avoiding aversive tasks for a short period of time, which in turn develops into procrastination. Previous studies have determined a relationship between procrastination and depression, possibly as a result, rather than a predictor, of depressed mental states [18]. One study has reported that both anxiety and depression increased students’ susceptibility to repeated negative thoughts about previous incidents, thus enhancing procrastination [19]. Fernie et al. have shown that depression is positively associated with dispositional avoidance (i.e., the tendency to avoid situations and problems) and procrastination, in a manner unrelated to anxiety [20]. This association may be partly explained by the symptomatology of depression, including diminished enjoyment in daily life events and difficulty concentrating, thereby impairing the capacity to initiate and complete tasks [21]. In summary, the effects of COVID-19 pandemic may be associated with anxiety and depression symptoms, which in turn are associated with procrastination. Therefore, we hypothesized that anxiety and depression symptoms would mediate the association between the effects of COVID-19 pandemic and adolescent procrastination, respectively.

Empirical studies have found that anxiety is a susceptibility factor for depression symptoms [22]. Beesdo et al. found social anxiety to be an antecedent variable for depression symptoms through a ten-year longitudinal study [23]. Other studies have also shown that anxiety is a risk factor for depression symptoms and plays a significant role in predicting the onset and development of depression symptoms in individuals [24], thus anxiety symptoms always precedes and has a significant positive predictive effect on depression [25]. Accordingly, we hypothesized that anxiety and depressed mood play a chain mediating role in the relationship between COVID-19 effects on adolescent procrastination.

Meanwhile, most of the previous studies are cross-sectional studies, which do not accurately reveal causality in a strict sense. For this reason, the present study proposes to construct a longitudinal chain mediation model based on the tracking data. Specifically, it is to comprehensively examine the chain mediation between anxiety and depression in the pathways of the effects of the COVID-19 pandemic and procrastination, and to explore the chain mediation between the effects of COVID-19 (T1) and procrastination (T4) through four times.

## Methods

### Sample and procedure

The participants in this study were 4,156 adolescents from 11 to 18 years old in the north of China, who were recruited through a convenient sample of key and non-key, public and private, and urban and rural middle and high schools. Four rounds of the survey were performed online through the survey website www.wjx.cn. The survey for this study was conducted at different time points, each reflecting different stages of the outbreak. Time 1 (T1, June 2020) marked the initial outbreak, when the entire province underwent a complete shutdown of work and school due to a major outbreak in the local area. This was followed by Time 2 (T2, December 2020), which took place 6 months later, coinciding with a slight relaxation of outbreak control measures. Moving forward, Time 3 (T3, August 2021) recorded a local outbreak with strict control measures in place. Finally, Time 4 (T4, October 2021) captured a small outbreak in a nearby area, prompting students to switch to online learning. A Chinese version of the questionnaires was available. We focused solely on participants who had at least one initial and any subsequent repeat measurements during the four surveys. A total of 925 (22.26%) participants completed all COVID-19 web surveys, whereas 1,251 (30.10%) completed only three web surveys, and 1,980 (47.64%) completed only two web surveys. All participants and their parents provided informed consent.

### Measures

#### Effects of the COVID-19 pandemic

Impact of Events Scale-Revised (IES-R) was completed by the participants [26]. IES-R is a commonly used tool to measure post-traumatic distress in people exposed to potentially distressing events [27]. IES-R includes 22 items, scored on a scale from 0 (not at all) to 4 (extremely). The total scores ranged from 0 to 88. The various difficulties reflected in the IES-R represent the symptoms of PTSD [28], including intrusive thoughts, arousal experiences and avoidance symptoms. The IES-R total score showed very good internal consistency, with Cronbach's alpha of 0.96.

### Procrastination

We calculated a composite score by summing items in the General Procrastination scale (GPS), a 20-item tool, which has been validated as a unidimensional measure of procrastination in multiple situations [29]. Responses are scored on a scale of 5 points ranging from “strongly disagree” to “totally agree.” A total score was determined for each wave of the pandemic (20–100). Cronbach’s alpha was 0.847 in this study.

### Anxiety symptoms

The seven-item Generalized Anxiety Disorder Scale (GAD-7) was derived from the diagnostic criteria for generalized anxiety disorder to screen and monitor anxiety severity [30]. It includes seven items, uses the same four ordinal response sets, and gives a severity score between 0 and 21. The GAD-7 has also been demonstrated to be valid and efficient in the Chinese population [31].

#### Depression symptoms

The nine-item Patient Health Questionnaire (PHQ-9) refers to the diagnostic criteria for major depressive disorder covered [32]. A total of nine items are included, each of which is rated in four ordinal response categories (from 0, not at all, to 3, nearly every day), denoting the extent to which the respondent has been bothered by these symptoms over the past 2 weeks. A severity score between 0 and 27 is determined. A Chinese version of the PHQ-9 has been developed and validated [33].

### Statistical analysis

First, descriptive statistics and bivariate Pearson’s correlation coefficients were analyzed in SPSS 22.0. Subsequently, chain mediating analysis was performed through structural equation modeling with Mplus 8.3 [34]. In the current study, isolated parceling was used to avoid the inflated measurement errors typically caused by multiple latent variables [35]. A value of *P* < 0.05 was considered significant. We performed chain mediation analysis estimation by using a regular bootstrapping sample approach [36] with 95% confidence intervals (CI) and 5,000 bootstrap samples. If 0 was not included in the 95% CI, the mediating effect was considered significant. Model fit indicators such as Chi-square/degree freedom (χ^2^/*df*), Comparative Fit Index (CFI), Tucker-Lewis Index (TLI), and Root Mean Square Error of Approximation (RMSEA) were utilized in the SEM [37]. The chi-square value is more affected by the sample size, so it needs to be combined with other indicators to make a comprehensive judgment. χ^2^/*df* < 5 is acceptable, 1–3 is good. CFI and TLI values more than 0.95 suggest good fit, whereas values greater than 0.90 imply adequate fit. RMSEA values of less than 0.05 are considered good, and values between 0.05 and 0.08 are deemed acceptable.

## Results

### Descriptive analysis

Table 1 provides an overview of our sample’s baseline characteristics. 4,156 participants; 2,109 were males (50.75%), and 2,047 were females (49.25%). The average age of the participants was 13.55 ± 1.18 years old, with a range of 11–18 years old. The number of middle school students, which accounted for 93.24% of the total, was 3875, while the number of high school students was 281 (6.76%). Of all the respondents, 3002 (72.23%) were only children, while 1154 (27.77%) had siblings. The majority (72.26%) of adolescents perceived their household economy as general and above, while only 4.41% perceived it as poor or very poor. The participants’ maternal education was mainly from middle school to undergraduate (91.16%). A few were in elementary school and below (4.96%) and graduate school and above (3.88%).

**Table 1:** Demographic and baseline information on participants included in the analyses

Variable	N	%	Variable	N	%
Gender			Presence of siblings		
Male	2109	50.75	No siblings	3,002	72.23
Female	2047	49.25	Siblings	1,154	27.77
Age			Household economy		
11–12	738	17.75	Very poor	40	0.96
13–14	2671	64.27	Poor	144	3.45
15–16	654	15.74	General	2,820	67.85
17–18	93	2.24	Good	933	22.44
Grades			Very good	219	5.30
Junior one	1,450	34.88	Maternal education		
Junior two	1,579	38.00	Primary or below	206	4.96
Junior three	655	15.76	Middle school	960	23.10
Junior four	191	4.60	High school	897	21.58
Senior one	269	6.47	Junior college	907	21.82
Senior two	10	0.24	Undergraduate	1,025	24.66
Senior three	2	0.05	Master or above	161	3.88

### Correlation analysis

The full results for all correlations, means and standard deviations are presented in Table 2. The effects of event (T1), anxiety symptoms (T2), depression symptoms (T3) and procrastination (T4) positively correlated with one another (all *P* < 0.01).

**Table 2:** Correlation analysis among variables

Variables	Mean ± SD	1	2	3	4
1 Effects of event (T1)	22.26 ± 18.01	1.00			
2 Anxiety (T2)	2.20 ± 3.70	0.28^a^	1.00		
3 Depression (T3)	3.22 ± 3.99	0.25^a^	0.33^a^	1.00	
4 Procrastination (T4)	49.82 ± 12.09	0.14^a^	0.23^a^	0.28^a^	1.00

^a^Indicates statistical significance (*P* < 0.01)

### Chain moderation model of anxiety (T2) and depression (T3) symptoms

**Fig. 1:**
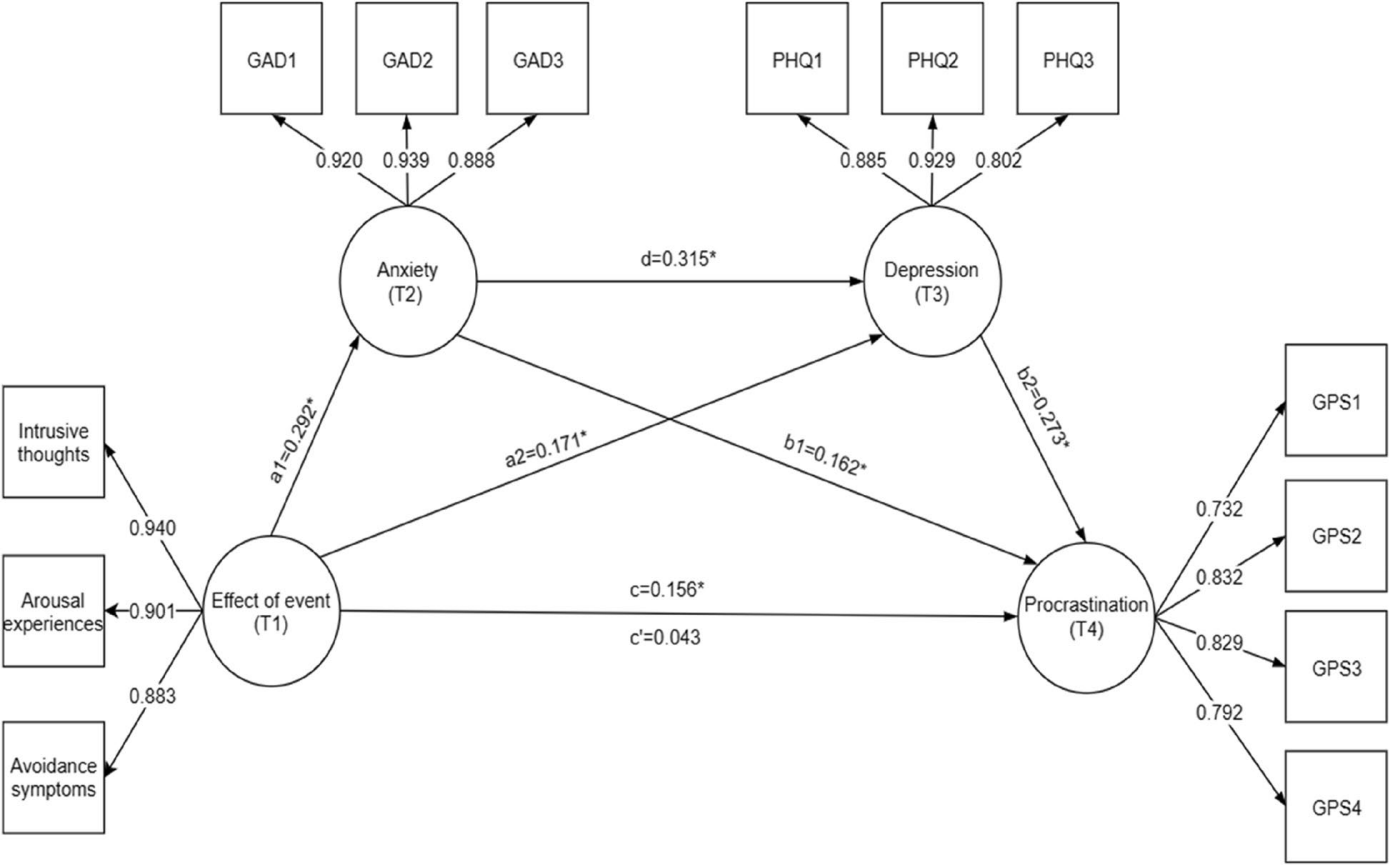
Chain mediating effects of anxiety (T2) and depression (T3) symptoms. Note: Path coefficients are shown as standardized regression coefficients (*P < 0.001)

The chain moderation analysis results of anxiety (T2) and depression (T3) symptoms on the effects of the COVID-19 pandemic (T1) on procrastination (T4) are shown in Fig. 1 and Table 3. The structural model fit the data well: χ^2^/*df* = 3.15; CFI = 0.995; TLI = 0.994; RMSEA = 0.023. As shown in Fig. 1, all factors loaded on the latent variables were significant (*P* < 0.001), thereby indicating satisfactory measurement of the model. The total effect (c) of the event (T1) on procrastination (T4) was significant (0.156). The direct effect (c’) of this model was not significant. The total standardized mediating effect value was 0.119. The ratio of the total standardized mediating effect to the total effect was 73.45%.

**Table 3:** Anxiety (T2) and depression (T3) symptoms in the mediation effect analysis

Pathway	Effect	SE	BootLLCI	BootULCI	Relative mediation effect
Total effect (c)	0.156	0.031	0.092	0.214	
Direct effect (c’)	0.043	0.033	-0.022	0.106	
a1	0.292	0.021	0.248	0.332	
a2	0.171	0.027	0.119	0.225	
d	0.315	0.035	0.247	0.386	
b1	0.162	0.039	0.080	0.237	
b2	0.273	0.035	0.200	0.341	
Indirect effects					
Total indirect effects	0.119	0.014	0.093	0.148	73.45%
Indirect 1	0.047	0.012	0.024	0.072	29.01%
Indirect 2	0.047	0.010	0.030	0.068	29.01%
Indirect 3	0.025	0.005	0.017	0.036	15.43%
Compare 1	0.000	0.008	-0.017	0.016	
Compare 2	0.011	0.007	-0.003	0.024	
Compare 3	0.010	0.005	0.002	0.020	

Abbreviations: *BootLLCI* Bootstrapping lower limit confidence interval, *BootULCI* Bootstrapping upper limit confidence interval, *SE* Standard error, Effect, regression coefficient. Indirect 1, effects of event (T1) → anxiety (T2) → procrastination (T4); Indirect 2, effects of event (T1) → depression (T3) → procrastination (T4); Indirect 3, effects of event (T1) → anxiety (T2) → depression (T3) → procrastination (T4). Compare 1: indirect effect 1–indirect effect 2; Compare 2: indirect effect 1–indirect effect 3; Compare 3: indirect effect 2–indirect effect 3

The mediating effect was composed of three indirect effects, and standardized results are shown. In indirect 1, anxiety (T2) mediated the effect of the event (T1) on procrastination (T4), with an effect value of 0.047. In indirect 2, the effects of the event (T1) on procrastination (T4) were mediated by depression (T3), with an effect value of 0.047. In indirect 3, the effects of the event (T1) on procrastination (T4) were mediated by both anxiety (T2) and depression (T3), with an effect value of 0.025. The ratios of the three indirect effects to the total effect were 29.01%, 29.01%, and 15.43% for indirect 1, 2, and 3, respectively. All three indirect effects reached the level of significance because the 95% CI of the above indirect effects did not contain the 0. Furthermore, unstandardized results indicated that the 95% CI comparing 1 and 2 included 0, thereby indicating that indirect 1 did not significantly differ from indirect 2 and 3. Comparison 3 indicated that the 95% CI for the difference between indirect effects 2 and 3 did not contain 0, thereby indicating that indirect effect 2 was significantly more effective than indirect effect 3.

## Discussion

Utilizing a longitudinal design, the present study aimed to explore the relationship between the effects of the COVID-19 pandemic and procrastination in adolescents and examine the underlying mechanisms of formation and exacerbation. Results from the study confirmed that the effects of the COVID-19 pandemic were positively predicted by adolescent procrastination. Additionally, anxiety and depression symptoms mediated the relationship between the effects of the COVID-19 pandemic and procrastination. Moreover, anxiety and depression symptoms also had chain mediation roles between the effects of the COVID-19 pandemic and procrastination.

Our findings suggest that the effects of the COVID-19 pandemic are positively associated with procrastination in Chinese adolescents and have a positive direct predictive effect. This is consistent with existing studies showing that negative life events were positively associated with procrastination among adolescents [38]. In addition, another study found that COVID-19 related emotional stress was related to bedtime procrastination among Chinese college students, which is similar to the results of the present study [39]. The ego-depletion theory suggests that controlling emotions may deplete limited internal cognitive resources, so high levels of stress may predict subsequent poor self-regulatory behavior [40]. Under COVID-19 pandemic long-term chronic stress, adolescents may need more time to rest and adapt from the stressful day, so tasks are procrastinated. Alternatively, procrastination behaviors result from adolescents' increased aversion to their own academic tasks in stressful situations. According to the self-regulatory failure theory, procrastination as a manifestation of self-regulatory failure may be due to low self-control in adolescents as a result of the effects of the COVID-19 pandemic [4].

This study suggests that anxiety and depression symptoms following the COVID-19 pandemic, affect the relationship between the effects of the COVID-19 pandemic and subsequent procrastination. On the one hand, the results suggest that adolescents who are more affected by the COVID-19 pandemic are more likely to be anxious, which contributes to their procrastination. The relevance of considering COVID-19 as a traumatic event and psychological trauma as a negative predictor of adolescent mental health has been confirmed in a cross-sectional study [41]. In fact, anxiety symptoms typically continue throughout a person's life, accompanied by avoidance behaviors. As psychological and social stimuli diminish, avoidance behaviors may lead to a rapid depletion of energy, which in turn leads to a decrease in behavioral control. On the other hand, the results suggest that adolescents who are more affected by the COVID-19 pandemic are by a stronger depressive mood, which contributes to their procrastination. A lagged daily diary analysis has indicated that negative emotions motivate procrastination behaviors: students have reported more procrastination behaviors after experiencing high levels of negative emotions [42]. Moreover, higher levels of depression have been found to be associated with more procrastination and to postpone the initiation of performing duties, in agreement with our results [43]. The effects of the COVID-19 pandemic on procrastination are mechanistically similar to the effects of depression and anxiety, and the negative emotions that are induced in a stressful situation can influence adolescents' personal behavior [38]. The current findings provide a reminder that symptoms of negative emotion should be brought to the attention of parents and teachers during the pandemic, and proactive and effective interventions should be implemented.

In addition, the study also found chain mediation effects of anxiety (T2) and depression (T3) symptom on the relationship between them. The longitudinal study found a significant specific mediating effect of the effects of the COVID-19 pandemic (T1) on procrastination (T4) through anxiety (T2) and depression (T3) symptoms effects. This suggests that the effects of the COVID-19 pandemic affect adolescents' depressed mood through increased levels of anxiety, which is ultimately associated with increased procrastination behavior. Previous studies have mostly been cross-sectional studies about the effects of negative life events or COVID-19-related emotional stress on procrastination [38, 39]. The present study bridges the gap between previous studies by exploring the causal relationship between the effects of the COVID-19 pandemic and procrastination through a longitudinal design, expanding the research in this area, and verifying the mediating role of anxiety and depressive symptoms between the effects of the COVID-19 pandemic (T1) and procrastination (T4), which suggests that an increase in anxiety symptoms can contribute to the level of procrastination, but this process may occur through an increase in depression symptoms. It can be seen that depression is a more direct influence on procrastination than anxiety, which further suggests a mechanism by which the effects of the COVID-19 pandemic influence procrastination levels.

### Limitations

This study has several limitations. The study population was selected using convenience sampling, which introduces significant sampling bias and limitations in sample representativeness. Therefore, it is not easily generalizable to the overall population. Similarly, the current study focuses only on middle and high school students in China under the COVID-19 pandemic, and it is not clear whether the results can be generalized to other national cultures or other contextual factors or adult populations. It should be feasible to replicate the methods used here in other settings to determine if the same type of categorization can be done elsewhere. The study’s longitudinal design had a relatively short time period of 16 months. A longer time period would aid in examining the causal sequence, and identifying trajectories and consequences in future investigations. Several other variables, such as behavioral habits and parenting styles, may also influence adolescents’ procrastination status. Further research may include these variables to control for their potential effects.

## Conclusions

Although there are some limitations to our study, it is also instructive for the problems that exist in modern life. The results of this study suggest that the effects of the COVID-19 pandemic can influence procrastination behavior by triggering anxiety and depressive symptoms in Chinese adolescents. Based on our findings, parents and schools can take several measures to reduce the impact of sudden stressful events similar to the COVID-19 pandemic on adolescents, such as conducting health education, providing psychological counseling services, and enriching extracurricular activities. These measures can help to further reduce negative emotions such as anxiety and depression in adolescents and ultimately improve adolescent procrastination.

## Data Availability

The datasets used and/or analysed during the current study available from the corresponding author on reasonable request.

## Abbreviations

COVID-19: Coronavirus Disease 2019
PTSD: Post-Traumatic Stress Disorder
IES-R: Impact of Events Scale-Revised
GPS: General Procrastination Scale
GAD-7: Seven-items Generalized Anxiety Disorder Scale
PHQ-9: Nine-items Patient Health Questionnaire
CI: Confidence Intervals
CFI: Comparative Fit Index
TLI: Tucker-Lewis Index
RMSEA: Root Mean Square Error of Approximation
